# Health-related quality of life, continuity of care and patient satisfaction: long-term outcomes of former patients of the Tuebingen Transition Program (TTP) – a retrospective cohort study

**DOI:** 10.1186/s12969-022-00776-6

**Published:** 2022-12-27

**Authors:** Luca Samuel Boeker, Jasmin Beate Kuemmerle-Deschner, Sebastian Jonas Saur, Jens Klotsche, Gabriele Erbis, Sandra Hansmann

**Affiliations:** 1 Department of Pediatrics, Division of Pediatric Rheumatology and autoinflammation reference center Tuebingen (arcT), University Hospital Tuebingen, Tuebingen, Germany; 2 Centre for Interdisciplinary Clinical Immunology, Rheumatology and Autoinflammatory Diseases and Department of Internal Medicine II (Oncology, Hematology, Immunology, Rheumatology, Pulmology), University Hospital Tuebingen, Tuebingen, Germany; 3 Programme area Epidemiology and Health Care Research, German Rheumatism Research Center Berlin and Leibniz Institute, Berlin, Germany

**Keywords:** Rheumatic diseases, Autoinflammatory diseases, Adolescence, Transition, Transfer, Long-term outcome, Quality of life, Patient satisfaction, Continuity of care, Juvenile idiopathic arthritis

## Abstract

**Background:**

A significant number of patients in pediatric rheumatology suffer from ongoing disease activity into adulthood and thus need to be transferred into adult care. Transition as a structured individual process of preparation and patient empowerment can reduce risks of adverse long-term outcomes. The aim of this study was to measure long-term transition outcomes such as health-related quality of life (HR-QoL), patient satisfaction, and continuity of care in former patients of the interdisciplinary Tuebingen Transition Program (TTP).

**Methods:**

In an iterative team process, a standardized questionnaire was developed including the EQ-5D-5L to measure HR-QoL, visual analogue scales to measure various items of patient satisfaction, further questions on continuity of care and physical activity and physician global assessment (PGA) to determine disease activity. HR-QoL and physical activity were compared to data from the average German population. Data was analyzed descriptively, and a logistic regression analysis was performed to identify possible predictive factors for negative outcomes.

**Results:**

Response rate was 28.8% (85/295), 70.6% were female and median age was 24.1 years. 70.6% were diagnosed with juvenile idiopathic arthritis (JIA). Overall, HR-QoL was high (79.8 on the EQ VAS), yet lower than in the average population. The study cohort was more physically active than the respective average age groups. Mean patient satisfaction with pediatric care (8.4; standard deviation (SD) 1.7) and with the transition program (7.9; SD 2.6) was higher than with adult care (7.7; SD 2.2). 76.5% of participants received regular rheumatologic care after transfer. After excluding all participants in remission, the drop-out rate was 4.7%. A low PGA at the time of transfer was associated with higher HR-QoL and patient satisfaction after transfer.

**Conclusions:**

HR-QoL of adult patients after successful transfer to adult rheumatology is reduced compared to the general population but physical activity and achievement of clinical remission could help to prevent negative long-term outcomes. Patient satisfaction and self-management of TTP patients were generally high, whereas youth-specific issues and their impact on the disease mandate greater attention. Treatment discontinuation rates were low and mostly due to remission. Further studies should focus on the identification of early predictors of long-term outcome to improve the process and outcome of transition.

**Supplementary Information:**

The online version contains supplementary material available at 10.1186/s12969-022-00776-6.

## Background

Pediatric rheumatic diseases comprise a broad spectrum of diseases with various clinical appearances and etiologies, including juvenile idiopathic arthritis (JIA) and its subgroups, autoinflammatory diseases, systemic connective tissue diseases (SCTD) and vasculitis. Although medical treatment of rheumatic diseases has developed and significant improvements have been made in recent years, more than half of all pediatric rheumatic disease patients and, excluding childhood restricted conditions, almost all patients with autoinflammatory diseases require ongoing anti-inflammatory treatment into adulthood and are at high risks of severe complications [1–4].

Therefore, structured and guided transition from pediatric to adult care is necessary once patients reach adolescence. Transition is not just an administrative process of gradually handing over care between pediatric and adult providers, but rather an individual process of young people gaining skills and accessing resources to manage the purposeful, planned transfer from a child-centred to an adult-centred health system [5]. Transfer in this respect is the final step of the transition process.

Without adequate support, young adults face an increased risk of poor outcome after the transfer from pediatric to adult care. Delay or discontinuation of transfer leads to higher disease activity and an increased risk of long-term morbidities, including cardiovascular events, osteoporosis and amyloidosis [3, 6]. The best possible preparation of patients is required to ensure a successful transfer and improved long-term quality of life. While there are standards and recommendations for establishing evidence-based transition programs [7], uncertainties about measuring long-term outcomes remain.

Many programs analyze satisfaction with the transition process as the main goal [8–11], but the focus of such programs is on long-term health and care improvement. An international group of interdisciplinary health professionals, patients and their families has developed consensus-based proposals for outcome indicators of a successful transition to adult health care. These can be summarized under the headings of individual outcomes, health care outcomes and social outcomes [12]. Another approach to evaluate transition outcome is the Triple Aim of the United States Institute for Healthcare Improvement: quality healthcare should consider three interdependent goals of improving care for individuals, improving population health, and reducing the per capita cost of care [13].

### Tuebingen transition program (TTP)

The Tuebingen Transition Program (TTP) is part of the Inflammation Center including the Center for Pediatric Rheumatology and the autoinflammation reference center Tuebingen (arcT) of the University Children’s Hospital, the Center for Interdisciplinary Clinical Immunology, Rheumatology and Autoinflammatory Diseases (INDIRA) and the Department for Internal Medicine of the University Hospital Tuebingen. With more than 20 years of experience, ongoing evaluation and supervised revision, it is a well-established program with a standardized transition process. The TTP is led by an interdisciplinary team, including pediatric and adult rheumatologists, psycho-social workers, physical therapists, nurses, and teachers.

Patients usually start in the TTP at age thirteen, depending on their individual maturity and disease activity. Regular interdisciplinary team meetings assess progress in independence and transition readiness in addition to disease progression. During the visits, the adolescent patients independently take over the interaction with the physicians - at first with the pediatric rheumatologist and with increasing age with the adult rheumatologist. Additionally, and in close cooperation with the psycho-social team, the focus of each visit is on promoting self-management, knowledge about the patient’s own disease and therapy and reflection of the individual progress, as well as on topics that are particularly relevant to adolescents. Other team members such as physiotherapists and teachers are also involved. At the end of each visit, parents are attending and included in the discussion about further treatment.

Regular readiness monitoring is carried out, and transfer is made in a remission phase if possible. In about one in three cases, referral to the rheumatology department of the Internal Medicine service takes place within the framework of the INDIRA network, especially for those patients with complex disease courses. The process of TTP is continuously evaluated and revised according to the latest standards and recommendations to ensure the best possible preparation for transfer.

The aim of this study was to assess the outcomes of TTP patients after transfer to adult care regarding health-related quality of life, patient satisfaction and continuity of care, and to identify potential early predictors of negative long-term outcome after transfer to adult care.

## Methods

### Assessment of the TTP – scoping review and questionnaire development

A scoping review of the PubMed database was conducted to identify relevant parameters for the evaluation of health care transition programs. Search terms included: “pediatric rheumatic disease”, “health care transition”, “long-term outcome”, “transfer”, “evaluation” and “transition program”. Studies were included if they focused on pediatric rheumatic diseases and either evaluated a transition program or addressed at least one health care transition outcome. In addition, a team of experts consisting of one adult and two pediatric rheumatologists, one psycho-social care provider, three patients currently in the program and three external reviewers provided input from their perspectives. Patients and external reviewers were selected to represent the study cohort in terms of age, gender, and medical knowledge. Patients were asked to rate comprehensibility and validity of the questions proposed by the expert team. In addition, they were asked to evaluate and assess the importance of certain components of transitional care in TTP, as well as various long-term outcomes considered for this study from their own experience and expectation. This influenced the selection of the outcome criteria and individual items of the standardized but not validated questionnaire, which was developed by the expert team in an iterative process.

This study was carried out in compliance with the Declaration of Helsinki and approved by the ethics committee of the Faculty of Medicine, University of Tuebingen (411/2020B0).

### Demographics and clinical characteristics

A single-center cross-sectional survey study of former patients of the TTP was performed. Patients were included if they were diagnosed with a rheumatic or autoinflammatory disease, were treated in the Inflammation Center for at least 2 years and during that time attended at least two visits in the TTP, one visit at the age of 17 or 18 years. All patients meeting these criteria between January 1, 2000, and December 31, 2019, were enrolled into the study. Patients who did not consent to participation or did not respond were excluded.

Demographic characteristics of the study cohort included age, gender, marital status, living situation and levels of education and employment. Clinical characteristics comprised diagnosis, therapy, Physician Global Assessment score (PGA) at time of transfer, and the need for physical and occupational therapy, each extracted from the medical chart at the time of transfer and self-reported by participants at the time this study was performed. The utilisation of offers from psycho-social care, clinic school, physiotherapy, patient education and transition camps was surveyed. Records on age at first visit, age at transfer and number of appointments at the TTP were collected.

All further demographic and clinical characteristics were extracted from the questionnaires and retrospectively at the time of transfer from the clinical documentation system ARDIS 2.0 (Arthritis and Rheumatology Documentation and Information System; axaris - software & systeme GmbH).

In order to estimate the demographic representativeness, demographic and clinical data were extracted from the medical chart of the non-responding cohort and were analyzed anonymously after the acquisition of data was completed.

### Individual outcome parameters

The health-related quality of life (HR-QoL) was assessed by the EQ-5D-5L, a validated instrument measuring the HR-QoL in five dimensions on a five-level Likert scale with equal spacing: mobility, self-care, daily activities, pain/discomfort, and anxiety/depression (1: none, 2: slight, 3: moderate, 4: severe, 5: extreme problems/unable to accomplish) [14]. From these scores, a personal health state as well as an index-value can be derived. Additionally, the EQ-5D-5L includes a visual analogue scale (VAS) on which participants are asked to express their current health status on a scale from 0 (“the worst health you can imagine”) to 100 (“the best health you can imagine”) [15]. The results were then compared to the average German population [16]. Furthermore, physical activity was assessed as another indicator for population health in the study cohort and compared to data from the German Health Interview and Examination Survey for Adults DEGS1 [17].

### Social outcome and patient satisfaction

Experience of care and patient satisfaction were categorized into several individual items related to pediatric, transitional, and adult care, with a focus on the quality of the transition process or long-term outcome after transfer. In the process of categorizing patient satisfaction, a standardized readiness checklist regularly used in the TTP as well as two similar questionnaires on patient satisfaction in health care transition were considered [10, 18]. All items were rated individually on a VAS from lowest (= 0) to highest possible satisfaction (= 10). After evaluating the first round of questionnaires, participants who had agreed to answer further questions were re-contacted and asked them to provide more detailed information on patient satisfaction in relation to the transition process and preparation for transfer. In accordance with Stringer et al., patient satisfaction scores > 7 were defined as “high”, ≤7 and ≥ 5 as “moderate” and <  5 as “low satisfaction” [18]. In addition, questions were asked about the patients’ experiences in adult care.

Further social outcomes were assessed regarding the influence of the COVID-19 pandemic on the current life situation and the influence of the disease on relationships and leisure time and patients’ social networks.

### Health care outcome

Continuity of care as an indicator of health care costs was assessed using self-reported information on the form and frequency of adult care, discontinuation and disruption of care, changes in diagnosis and treatment and hospital admissions. A comprehensive comparison was made between the two groups of participants with and without continued care after transfer with continued care being defined as self-reported initial regular appointments in adult rheumatological care, in order to identify differences that might serve as possible predictive factors for an unsuccessful transfer.

### Exploration of factors correlating with a negative long-term outcome and statistical analysis

Possible factors correlating with a decreased outcome in HR-QoL and patient satisfaction were explored.

Data were collected using Microsoft Excel for Mac version 16.54. For analysis, data were transferred to IBM SPSS Statistics version 27.0.1.0. and primarily analyzed descriptively. In addition, linear regression analyses, Mann-Whitney U tests and Kruskal-Wallis tests were performed to identify relevant correlates between demographic, clinical or other variables and long-term outcomes. These were complemented by suitable analyses of power and effect size. Medical therapies were grouped according to common practice.

## Results

### Assessment of the TTP - scoping review and questionnaire development

The following issues were identified through the literature review and from the expert team: quality of life, utilization of care, satisfaction with care, self-management, knowledge about one’s condition and therapy, continuation of care after transfer, avoidance of unnecessary hospitalizations after transfer, relationship to adult caregiver, personal relations and social network. Those were transferred into a standardised questionnaire by the expert team and covered demographic and clinical data characteristics after transfer, individual outcome parameters including HR-QoL measured with the EQ-5D-5L, health care outcomes, social outcomes, and patient satisfaction.

### Demographics and clinical characteristics

All former patients of the TTP were contacted. 85 of 295 returned the completed questionnaire, resulting in a response rate of 28.8%. Of the study participants, 60 were female (70.6%) and the median age was 24.1 years (range 19.1–40.5). The most common diagnosis was JIA (70.6%), followed by SCTD (9.4%) and autoinflammatory diseases (7.1%). At the time of transfer, 27% of participants (23/85) did not receive any prescribed medication. Almost a third each received basic antirheumatic therapy (28.2%) and biologicals including Janus kinase (JAK) inhibitors (32.9%). The mean PGA at the time of referral was 1.01 (SD 1.15) and more than three out of four participants (65, 76.4%) had achieved a state of clinical remission defined as PGA ≤1. Patients were treated for a median duration of 3.4 years in TTP, median age at first visit was 15.2 years and median age at transfer was 18.5 years. While the study cohort had a high level of education overall, 42.4% of participants (36/85) had not completed vocational training at the time the study was conducted. More than 95% (47/49) of the participants who had completed vocational training reported that they were currently employed. Utilization of the specific services offered within the framework of the TTP varied from 44.7% using psycho-social services to only 2.4% using more extensive schooling opportunities. Detailed demographic and clinical data are shown in Table 1.

**Table 1:** Demographics and clinical characteristics of the study cohort

Variable	Result
Age in years, median (range)	24.1 (19.1–40.5)
18–24 years, n (%)	49 (57.6)
25–34 years, n (%)	33 (38.8)
35–44 years, n (%)	3 (3.6)
Time after transfer in years, median (range)	6.0 (0.1–16.4)
Number of days between last visit in TTP and first visit in adult care, median (range)	128 (13–983)
Gender, m:f, n (%)	25:60 (29.4:70.6)
Diagnosis at time of transfer, n (%)	
JIA, overall	60 (70.6)
sJIA	2 (2.4)
OA extended	3 (3.5)
OA persistent	10 (11.8)
PA (RF-)	23 (27.0)
ERA	14 (16.5)
Psoriatic Arthritis	5 (5.9)
JIA, other	3 (3.5)
Autoinflammatory Disease	6 (7.1)
Systemic connective tissue disorders	8 (9.4)
Other	11 (12.9)
Medical therapy at time of transfer, n (%)	
None	23 (27.0)
NSAID therapy (on demand)	2 (2.4)
NSAID therapy (daily regimen)	5 (5.9)
Basic therapy	24 (28.2)
Biologicals and JAK-inhibitors	28 (32.9)
Steroids	2 (2.4)
Missing	1 (1.2)
PGA at time of transfer	
Mean (SD)	1.01 (1.15)
Median (range)	1 (0–5)
PGA = 0, n (%)	33 (38.8)
PGA = 1, n (%)	32 (37.6)
PGA = 2, n (%)	12 (14.1)
PGA = 3, n (%)	4 (4.7)
PGA = 4, n (%)	2 (2.4)
PGA = 5, n (%)	2 (2.4)
Utilization of the TTP in general	
Age at first TTP visit in years, median (range)	15.2 (11.2–18.7)
Age at transfer in years, median (range)	18.5 (17.0–20.7)
Duration of TTP care in years, median (range)	3.4 (0.4–7.1)
Number of TTP visits, median (range)	7.0 (2–18)
Utilization of additional services of the TTP	
Psycho-social services, n (%)	38 (44.7)
Clinic school, n (%)	8 (9.4)
Disease-related schooling opportunities, n (%)	2 (2.4)
Transition camp, n (%)	4 (4.7)
Physical therapy, n (%)	28 (32.9)
Level of school graduation, n (%)	
Certificate of Secondary Education (Hauptschulabschluss)	8 (9.4)
General Certificate of Secondary Education (Mittlere Reife)	11 (12.9)
Advanced technical college entrance qualification (Fachhochschulreife)	9 (10.6)
General qualification for university entrance (Allgemeine Hochschulreife/Abitur)	56 (65.9)
Other	1 (1.2)
Completed vocational training, n (%)	
Yes	49 (57.6)
No	36 (42.4)
Current employment, n (%)	
Yes	47 (55.3)
No	34 (40.0)
Missing	4 (4.7)
Marital/family status, n (%)	
Single	74 (87.1)
Married	9 (10.6)
Other	2 (2.4)
Domestic circumstances, n (%)	
Living alone	21 (24.7)
At the parents’ home	31 (36.5)
Shared apartment	11 (12.9)
Living with the partner or family	18 (21.2)
Other	3 (3.5)
Missing	1 (1.2)

Legend: *m* Male, *f* Female, *NSAID* Nonsteroidal anti-inflammatory drugs, *PGA* Physician’s Global Assessment Score (0: no disease activity; 10: highest possible disease activity), *TTP* Tuebingen transition program

In comparison to the non-responding cohort, participants were significantly younger and had an insignificantly lower PGA at the time of transfer. Autoinflammatory diseases and SCTD were more frequent in non-respondents. Patients who didn’t respond were also more likely to be treated with steroids (Supplement 1).

### Individual outcome parameters

#### Health-related quality of life

The majority of the study cohort reported no difficulties in each category of the EQ-5D-5L while three out of four participants indicated no more than slight problems in any category. Participants rated their own current health status at an average of 79.8 (SD 19.0) on the EQ VAS.

**Fig. 1:**
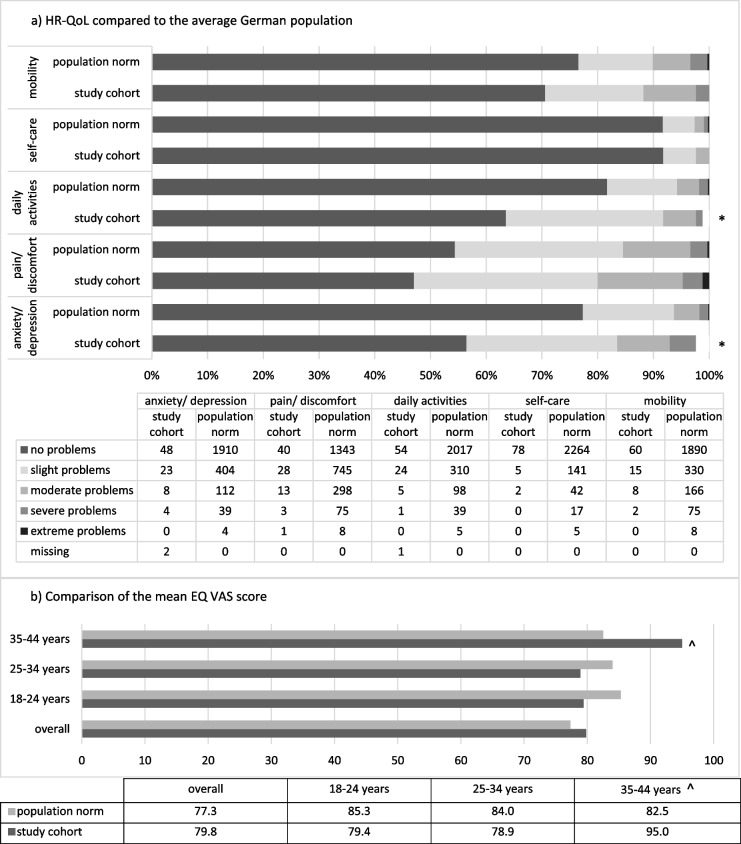
Comparison of the mean EQ VAS score and dimensions with the average German population. **a** HR-QoL compared to the average German population. **b** Comparison of the mean EQ VAS score. Legend: **a** The percentages of all participants reporting different degrees of problems in the different dimensions of the EQ-5D-5L, which are lined up vertically, sum to 100%. These are compared between the study cohort and the average population. * marks significant differences between study cohort and average population. **b** Mean scores of the EQ-VAS (0–100) overall and divided by the age groups represented in our study cohort. The mean total population score is lower than the mean scores of the age groups shown because older age groups were included that are not represented in this review. ^ marks age group in which the study cohort is only represented by 3 persons

Compared to the average German population, the study cohort presented significantly more problems with daily activities and anxiety/depression (*p* <  0.005), while it was similar to the population in terms of self-care (Fig. 1a). The study cohort showed a lower mean score of the EQ VAS compared to the average German population. In the older age group, this was reversed. Due to the small number of participants, no significance test was conducted (Fig. 1b).

#### Physical activity

**Fig. 2:**
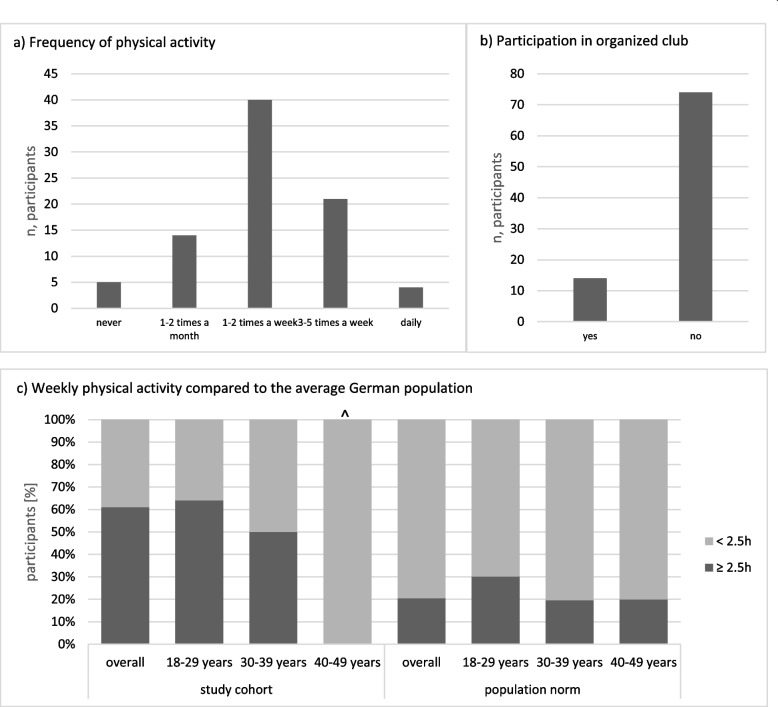
Physical activity in the study cohort*.* **a** Frequency of physical activity. **b** Participation in organized club. **c** Weekly physical activity compared to the average German population. Legend: a) The figure shows the number of participants who reported each of the listed frequencies of physical activity (*n* = 84 due to one missing data). **b** The figure shows the number of participants who are physically active in organised sports clubs (*n* = 84 due to one missing data). **c** Each column represents 100% of participants either overall or in the respective age group. They are divided into participants who reported weekly physical activity of 2.5 hours or more (lower dark bar) and those who reported less weekly physical activity (upper light bar). ^ marks age group in which the study cohort is only represented by 1 person

The reported average duration of physical activity per week was 3.4 hours (SD 2.9) and ranged from zero to 14 hours per week. Fourteen participants (16.5%) stated that they were active in an organised sports club, only five that they were not physically active at all. More than three out of four respondents mentioned being physically active at least once a week. Figure 2a, b gives an overview of the frequency of physical activity and participation in organized sports.

In the study cohort, 61.0% reported at least 2.5 hours of physical activity per week in comparison to only 20.4% of the average German population. This difference was seen in both the 18–29-year-olds (64.1% vs. 30.2%) and the 30–39-year-olds (50.0% vs. 19.6%) (Fig. 2c).

### Social outcome and patient satisfaction

Overall satisfaction as well as satisfaction with relevant components of pediatric, transitional, and adult care was generally high. All items, except the two adolescent-specific topics and preparation for transfer, reached a mean score >  7. In general, the mean scores ranged from 5.38 to 9.03 (mean: 7.63). Participants were most satisfied with the way their individual questions were addressed (mean: 9.03), their knowledge about their own disease (mean: 8.51) and self-management skills (mean: 8.46) at the time of transfer. Along with the satisfaction with pediatric care in general (mean: 8.42), these were the items with the highest rate of highly satisfied participants. Overall satisfaction decreased from an average of 8.42 in pediatric care to an average of 7.65 in adult care. Table 2a shows detailed results for patient satisfaction in each item.

**Table 2:** Social outcome and patient satisfaction

**a) Satisfaction with …**	**Mean score (SD)**	**Missing n (%)**	**> 7** **n (%)**	**≤ 7 - ≥ 5** **n (%)**	**< 5** **n (%)**	**n**
**Components of care before transfer (TTP)**						
Paediatric care in general	8.42 (1.68)	1 (1.2)	71 (83.5)	11 (12.9)	2 (2.4)	85
Preparation for transfer	6.78 (3.02)	5 (5.9)	48 (56.5)	17 (20.0)	15 (17.6)	85
TTP in general	7.90 (2.55)	4 (4.7)	62 (72.9)	10 (11.8)	9 (10.6)	85
How alcohol, nicotine and drugs were addressed	6.29 (2.91)	1 (2.9)	13 (37.1)	12 (34.3)	9 (25.7)	35^a^
How sexuality, contraception and family planning were addressed	5.38 (2.85)	1 (2.9)	8 (22.8)	17 (48.6)	9 (25.7)	35^a^
How participants’ individual questions were addressed	9.03 (1.20)	0 (0.0)	30 (85.7)	5 (14.3)	0 (0.0)	35^a^
How participants were involved in decision-making	8.17 (1.89)	0 (0.0)	24 (68.6)	10 (28.6)	1 (2.8)	35^a^
Knowledge about one’s disease	8.51 (1.85)	0 (0.0)	27 (77.1)	6 (17.2)	2 (5.7)	35^a^
Knowledge about one’s therapy and possible side-effects	7.97 (2.07)	0 (0.0)	23 (65.7)	9 (25.7)	3 (8.6)	35^a^
One’s self-management skills	8.46 (2.12)	0 (0.0)	29 (82.9)	4 (11.4)	2 (5.7)	35^a^
**Components of care after transfer**						
Adult care in general	7.65 (2.22)	0 (0.0)	44 (67.7)	13 (20.0)	8 (12.3)	65
Waiting time for an appointment	7.58 (2.37)	0 (0.0)	43 (66.2)	12 (18.5)	10 (15.4)	65
Waiting time at the appointment	7.11 (2.50)	0 (0.0)	41 (63.1)	13 (20.0)	11 (16.9)	65
Interaction with doctors	7.45 (2.63)	0 (0.0)	41 (63.1)	12 (18.5)	12 (18.5)	65
Prescription of further therapeutic options	7.75 (3.09)	6 (9.2)	43 (66.2)	8 (12.3)	8 (12.3)	65
**b) Effect of …**		**Missing** **n (%)**	**Negative** **n (%)**	**No effect** **n (%)**	**Positive** **n (%)**	**n**
the COVID-19 pandemic on the current life situation, n (%)		2 (2.4)	46 (54.0)	27 (31.8)	10 (11.8)	85
the disease on personal relationships, n (%)		3 (3.5)	22 (25.9)	54 (63.5)	6 (7.1)	85
**c) Questions**		**Missing** **n (%)**	**No** **n (%)**	**Yes** **n (%)**		**n**
“Did your disease affect leisure activities at all?”		5 (5.9)	67 (78.8)	13 (15.3)		85
“Did you receive support in managing your condition from within your social environment?”		8 (9.4)	26 (30.6)	51 (60.0)		85
“Did you have enough time to ask all the questions that were important to you individually, during the first two visits in adult care?”		0 (0.0)	9 (13.8)	56 (86.2)		65
“Did you receive sufficient and intelligible answers?”		0 (0.0)	10 (15.4)	55 (84.6)		65
“Did you feel like you were taken seriously?”		0 (0.0)	10 (15.4)	55 (84.6)		65
“Were you involved adequately in decisions concerning your individual treatment?”		0 (0.0)	9 (13.8)	56 (86.2)		65

Legend: Patient satisfaction was measured on a 10 cm visual analogue scale (VAS) and differentiated in 3 groups (VAS > 7; VAS ≤ 7 - ≥ 5; VAS > 5). Totals vary due to some participants who never transferred to adult care and therefore did not answer the questions about the components of care after transfer

^a^These more detailed questions were only assessed by those participants who agreed to take an additional telephone survey, which contained more specific questions about general patient satisfaction

Concerning their experience in adult care, 86.1% (56/65) declared that they had enough time to ask all their individual questions and 84.6% (55/65) stated that they received sufficient and intelligible answers. 84.6% (55/65) felt that their concerns were taken seriously by their practitioner and 86.2% (56/65) specified that they had been involved adequately in decisions concerning their individual treatment.

More than half of the participants (46; 54.0%) stated that the pandemic had negative effects on their current life situation. While 10 participants (11.8%) reported positive effects, almost one third (31.8%) indicated that the pandemic did not affect their life situation in either way (Table 2b).

60% of participants (51/85) felt that they had sufficient support by their social environment, mostly through their family, partners or spouses and close friends. Still, one out of four respondents (22/85; 25.9%) indicated that their disease had negative effects on relationships towards friends and family. Six participants (7.1%) experienced positive effects, while 63.5% (54/85) did not obtain any effects of their disease on social relationships (Table 2b, c).

### Health care outcome

#### Continuity of care

**Fig. 3:**
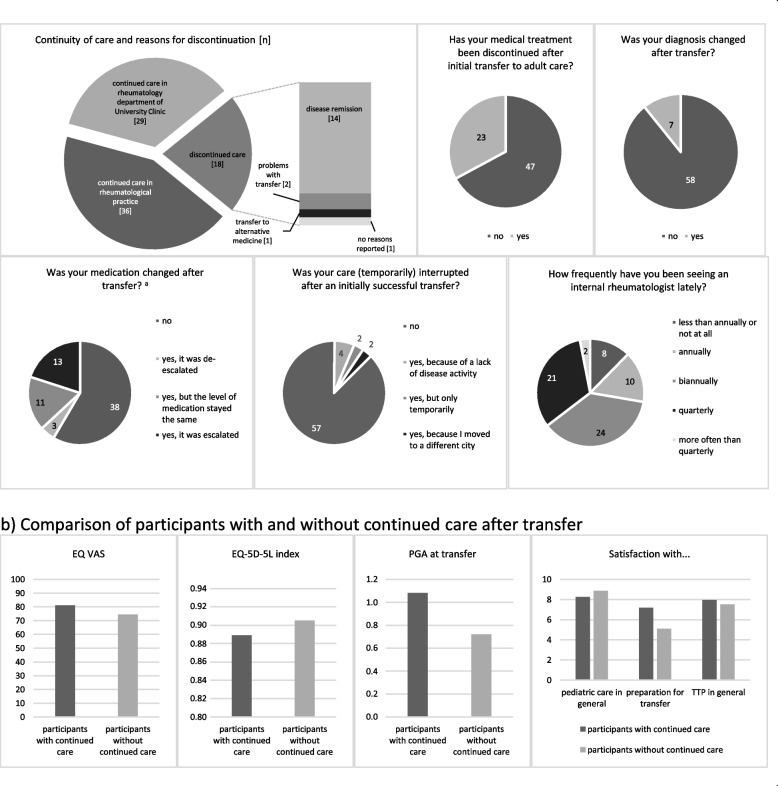
Continuity of care*.* **a** Descriptive results of continuity of care. **b** Comparison of participants with and without continued care. Legend: **a** Descriptive results on continuity of care. The first graph shows the absolute numbers of participants with and without continued care after transfer and the reasons given for discontinuation of care (*n* = 83 due to two missing data). The following charts refer to the 65 patients who initially reported a successful transfer to adult rheumatology. These charts show the number of patients with a discontinuation of care after initial transfer to adult rheumatology, reasons for discontinuation of care, changes in diagnosis or medication after transfer and the current frequency of visits to adult rheumatology. a We ranked basic therapy as level 1, biologicals and JAK-inhibitors as level 2, and steroids as level 3. NSAIDs were ranked as level 0, whether they were prescribed to be taken daily or on demand. **b** Comparison of mean scores of relevant parameters between participants who continued to receive care after transfer and those who did not. Higher scores on the EQ VAS and lower EQ-5D-5L index scores indicate better health-related quality of life, lower PGA scores indicate less disease activity

More than three out of four participants (65/85; 76.5%) stated that they continued to receive rheumatological care after leaving the TTP. The majority were treated in rheumatology practices (36/65; 55.4%), while the remaining participants (29/65; 44.6%) had been referred to the internal rheumatology department of the University Hospital Tuebingen. Of the 18 participants whose rheumatological treatment had been discontinued, 14 cited a lack of disease activity as the main reason. Only two participants reported problems with referral that led to the discontinuation of rheumatological care (Fig. 3a).

Of the 65 participants who reported continuation of care after the transfer, eight stated that care had been disrupted afterwards. While four of them cited lack of disease activity as the reason and for two other participants it was only a temporary interruption, two participants stated that they had not found a new rheumatologist after moving to another town. Of the 65 participants who initially received continued care, 12.3% (8/65) reported that they were seeing their rheumatologist less than once a year, 15.4% (10/65) reported a yearly, 36.9% (24/65) a six-monthly frequency of visits, while 33.8% (23/65) reported four or more visits per year.

Twenty-three participants (23/65; 35.4%) indicated that their prescribed medication was discontinued after transfer to adult care. The most common reason for discontinuation was remission, followed by discontinuation at the patient’s request. In addition, 27 participants (27/65; 41.5%) reported that their prescribed medication was changed during adult care. Thirteen of them had their therapy escalated and three de-escalated. Seven participants reported that their diagnosis had been changed after transfer to adult care (Fig. 3a). In three cases, a stable remission occurred, leaving them with the diagnosis of “condition after” their initial pediatric rheumatic diagnosis. Three different patients had their diagnosis changed to the adult equivalent of their pediatric rheumatic diagnosis, e.g., from JIA to rheumatoid arthritis. One patient developed further symptoms, so his diagnosis was modified.

71.8% of participants (61/85) reported no hospitalisations after leaving the TTP. In total, participants reported nine hospitalisations due to rheumatic diseases after transfer to adult care. Reasons for hospitalisations relevant to this study were repeated inflammatory episodes, joint (knee and shoulder) and ophthalmologic complications.

#### Differences between participants with and without continued care

Participants with continued care showed a higher mean score on the EQ VAS for current condition, while in average scoring lower in the EQ-5D-5L index for HR-QoL. Participants who did not receive continued rheumatologic care had a lower PGA at time of transfer (0.72 compared to 1.08 for participants with continued care) and showed notably lower satisfaction with preparation for transfer (5.10) compared to participants with continued care (7.19). Further details can be found in Fig. 3b. Overall, continuity of care was associated with higher patient satisfaction in various categories (Supplement 2).

### Explorative analysis of factors correlating with a negative long-term outcome

#### Factors correlating with lower HR-QoL

Various items of patient satisfaction showed a significant correlation with HR-QoL. In every instance, lower satisfaction was associated with lower scores in the EQ-5D-index and the EQ VAS (Table 3a). In addition, female gender, higher disease activity at time of transfer as measured by the PGA, higher frequency of visits in adult care after transfer and lower frequency of physical activity were associated with significantly lower HR-QoL (Table 3b).

**Table 3:** Significant effects on HR-QoL and patient satisfaction

***a****: Significant effects of patient satisfaction on HR-QoL*				
**Dependent variable**	**Independent variable** Satisfaction with...	**beta-coefficient**		***p*-value**
EQ-5D-index	preparation for transfer	0.273		0.016
	how alcohol, nicotine and other drugs were addressed	0.476		0.005
	adult care in general	0.328		0.008
	waiting time for an appointment in adult care	0.287		0.020
	waiting time at an appointment in adult care	0.265		0.033
	interaction with the practitioner in adult care	0.282		0.023
EQ VAS	paediatric care in general	0.309		0.005
	preparation for transfer	0.427		< 0.001
	TTP in general	0.317		0.004
***b:*** *Various significant effects on HR-QoL*				
**Dependent variable**	**Independent variable**	**beta-coefficient**	**Eta-coefficient**	***p*-value**
EQ-5D-index	PGA at time of transfer	−0.362		< 0.001
	Frequency of visits in adult care	− 0.335		0.002
Problems with mobility	PGA at time of transfer	0.306		0.004
	Frequency of physical activity	−0.241		0.027
Problems with self-care	PGA at time of transfer	0.271		0.012
Problems with daily activities	Gender: male		0.276	0.011
	Frequency of visits in adult care	0.255		0.021
	Frequency of physical activity	−0.300		0.006
Pain/discomfort	PGA at time of transfer	0.286		0.008
	Frequency of visits in adult care	0.361		< 0.001
Anxiety/depression	Frequency of physical activity	−0.340		0.002
EQ VAS	Gender: male		0.234	0.033
	Frequency of physical activity	0.260		0.018
	Satisfaction with TTP in general	0.317		0.004
***c:*** *Various significant effects on patient satisfaction*				
**Dependent variable** Satisfaction with...	**Independent variable**	**beta-coefficient**		***p*-value**
how alcohol, nicotine and other drugs were addressed	PGA at time of transfer	−0.582		< 0.001
how sexuality, contraception and family planning were addressed	PGA at time of transfer	−0.461		0.032
adult care in general	PGA at time of transfer	−0.273		0.025
prescription of further therapeutic options	PGA at time of transfer	−0.344		0.042
preparation for transfer	Time passed since transfer	−0.340		0.002

Legend:a) Each specific category of patient satisfaction presented above showed a significant correlation with HR-QoL in a linear regression analysis. A more positive beta coefficient means a stronger positive correlation. For manageability, we have limited the HR-QoL in this presentation to the EQ-5D index and the EQ VAS and left out the correlations between patient satisfaction and the five individual dimensions of the EQ-5D-5L

b) The EQ-5D index, the dimensions of the EQ-5D-5L and the EQ VAS showed significant correlations with various factors outside patient satisfaction. More positive beta coefficients signify a stronger positive correlation, while negative beta coefficients signal negative correlations. In cases where the beta coefficient is indicated, the indicated group had a significantly higher HR-QoL than the non-indicated opposite group

c) Patient satisfaction showed correlations with several factors listed above. More negative beta coefficients mean a stronger negative correlation. A higher ‘time since transfer’ means that participants attended the TTP longer ago than others, so their referral is further in the past

#### Adjusted and weighted prevalence/factors correlating with lower patient satisfaction

A higher PGA score at time of transfer correlated with lower scores in four different items of patient satisfaction. More time passed since transfer was associated with lower satisfaction with preparation for transfer (Table 3c).

## Discussion

This is one of the first studies to comprehensively evaluate a structured transition program based on all three aspects of the Triple Aim [13]. The response rate in this study was 28.8%, however, it provides detailed insights into the long-term outcomes of a number of TTP patients after transfer to adult rheumatology care and indicates possible predictors of unsuccessful transition. The majority of the study cohort expressed high and stable HR-QoL, and most of them were very satisfied with pediatric and adult care, and with TTP in general. After the transfer, most participants had their first regular follow-up within 4–5 months and rheumatological care continued regularly. The results give the idea that physical activity and reaching clinical remission could help to prevent negative long-term outcomes after transfer.

HR-QoL as the primary outcome parameter to evaluate long-term outcome was overall high among respondents. There were only minimal to moderate difficulties in the individual categories of the EQ-5D-5L. In comparison to the average German population the study cohort reported slightly lower HR-QoL on the EQ VAS [19] and significantly more problems with daily activities and anxiety/depression, while showing no difference in terms of self-care [16]. High PGA scores at the time of referral and low physical activity correlated with lower long-term HR-QoL.

Although patients with rheumatic diseases are found to have a lower HR-QoL [20], prospective studies were able to demonstrate that transition programs can improve quality of life (QoL) of participants [21], even in comparison to control subjects [22].

The difference in the individual categories of the EQ-5D compared to the average German population was more pronounced in a study by Barth et al. that observed former patients of a large German clinic with JIA, especially for self-care [23]. Barth et al. used the EQ-5D-3L which showed a stronger ceiling effect than the EQ-5D-5L. That should make the latter more reliable for detecting differences compared to population norms.

The better outcome in this study cohort may be partly due to the high number of participants who had reached a state of remission before transfer to adult care. This is supported by the strong correlation between a lower PGA at the time of transfer and a higher long-term HR-QoL. The TTP also emphasizes the importance of physical activity for the well-being of patients with rheumatic diseases found in multiple studies [24–26]. Thus, the correlation between higher frequency of physical activity and higher HR-QoL can also be an indication of the effectiveness of the TTP for the responding cohort.

Most respondents reported a negative influence of the COVID-19 pandemic on their current life situation. However, this study did not find significant effects on HR-QoL. Although the associations remain very complex, many studies similarly found that the pandemic had negative influences on physical and psychological well-being in the general population [27–29].

Although the quality of life of former pediatric rheumatology patients remains impaired, this study found promising signs that could support the known positive effects of a good health care transition program on improving long-term outcomes.

Respondents indicated a high patient satisfaction, although it decreased after transfer to adult care. While participants indicated high satisfaction with the TTP in general, satisfaction with the communication of topics relevant to adolescents, such as the impact of drugs and sexuality on living with a rheumatic disease, was rated lower. In contrast, the items “how participants individual questions were addressed”, disease-knowledge and self-management were rated above average.

Patient satisfaction is an important indicator for effective high-quality transition [30] and the most commonly studied issue, although validated instruments in all languages remain lacking [31, 32]. German instruments of appropriate length were still being developed and validated while this study was conducted [33]. The longer version of this instrument unfortunately did not seem fitting for this study since it surveyed topics that do not apply to the process and cohort of the TTP, e.g., working environment. Still, the questions asked in this study have been used in different studies [10, 18] and in the ongoing evaluation of patients’ readiness for transfer in the TTP. Overall satisfaction with transition, respectively transition clinics, was slightly higher than in comparable studies [18, 34]. Like Stringer et al. [18], this study found a similar variability in satisfaction with single items of the transition process and likewise low scores in adolescence-specific topics. Klein et al. [10] have described a decline in overall patient satisfaction after the transfer to adult care, which was observed to a lesser extent in this cohort. The relevance of achieving self-management skills in preparation for transfer is widely accepted [7, 30]. Walter et al. have found a connection between successful transition and high self-management skills [34]. Thus, the high patient satisfaction with self-management skills in this study could serve as an additional indicator for a successful long-term outcome of the TTP.

Continuity of care is a critical aspect of successful referral [30]. Overall, this study found high follow-up rates in the cohort within the TTP, while 21.2% of patients reported not seeing an adult rheumatologist regularly. However, the distinct data for reasons for discontinuation of care indicated that a large proportion of patients were in stable remission and did not require continuous care. Apart from patients in remission, 4.7% discontinued treatment in adult medicine due to referral problems.

Similar studies have found lower as well as comparable rates of successful transfer (range: 48.0–74.0%) [10, 18, 35] while Walter et al. reported drop-out rates of only 5.1% at most [34]. The authors partly attributed these numbers to the habit of transferring patients within the same hospital following the EULAR and PReS recommendations [7]. In the TTP, this is handled similarly for patients with more severe courses of disease. Furthermore, the TTP tries to individualise the timing of transfer in close cooperation with patients, parents, the medical team, and psycho-social workers according to patients’ transfer readiness and disease activity. This relates to the large difference in follow-up rates between patients with and without disease activity at the time of their last visit in the TTP. The data of this study confirmed the assumption of Hilderson et al. that remission is the most likely reason for the lack of follow up [36]. To some extent, this is comparable to the results of Hazel et al. [35], who also reported a significantly lower transfer rate for patients in remission.

When drop-out due to remission is disregarded, a high follow-up rate as a result of successful transition can be observed and is an affirmation of the notion to individualise the timing of transfer.

As mentioned above, this study is limited by a response rate of 28.8%. Thus, statistical power is limited, and bias is possible. For one aspect, it can be assumed that patients who had a better experience in the TTP might have been more likely to respond to the invitation to participate in this study than those who have no good memories of their time in the TTP. Likewise, responsiveness could be considered as an indicator of a more successful transition in a sense that a better self-management can be associated with a higher sense of responsibility when being invited to such a study. Thus, it is possible that the non-responsive cohort might have indicated less successful long-term transition outcomes than the responders. In any case, the generalizability of the data collected in this study is limited.

As assumed in advance, responsiveness decreased with time passed since patients were transferred resulting in significantly lower age in participants compared to the non-responsive cohort. Still, it was an intentional decision to include a wider time span to increase the number of participants, especially since response rates in comparable studies are similar to this one. Except for the age difference, no significant demographic differences were found between participants and the non-responsive cohort. In this aspect, the study cohort tolerably represents the overall patients of the TTP.

Limitations also resulted from the study design of a cross-sectional patient survey, as the data obtained in such studies cannot prove causal attribution. Due to the individual time period of termination of treatment in the TTP and the completion of the survey between 1 month and 16 years after transfer, it cannot be excluded that the individual perception of the TTP has changed over time. For further research, it is recommended to develop a prospective approach with defined study data before and after referral.

The criteria for evaluating long-term outcomes in transition are complex constructs, and often validated measurement tools in the patients’ language are lacking. This limits the power and validity of such studies.

## Conclusions

This study contributes to further optimize transition and promote long-term health of pediatric rheumatology patients, measuring individual, social and health care outcomes. While HR-QoL remained lower compared to the average German population, physical activity in the study cohort was higher than in the average population and exceeded WHO recommendations. Respondents indicated high patient satisfaction and self-perceived self-management skills and felt well informed. Certain adolescence-specific issues should be given even more attention. This study found a high rate of continued care after transfer among respondents, fortunately with remission being the main reason for discontinuation. The results of this study seem to affirm the suggestion to individualize the timing of transfer in order to refer adolescents with the lowest possible disease activity. Furthermore, the importance of physical activity for the long-term HR-QoL of rheumatologic patients seems to be supported in this cohort. Overall, there were indications for positive effects of a structured health care transition program with a multidisciplinary approach including psycho-social care and physiotherapy.

Further prospective studies should more thoroughly investigate possible predictive factors of long-term outcome, focusing on readiness for transfer, patient activation and adherence and age at entry into the transition program as potential factors.

## Supplementary Information

**Additional file 1. Supplement 1.** Comparison of basic demographic and clinical characteristics between responsive and non-responsive cohort. Legend: Listed are some key demographics and clinical data of the respondents compared to the non-respondents of this study. Overall, respondents were slightly younger, and subgroups of JIA were more common as well as treatment with biologicals. **Supplement 2:** Significant differences between participants concerning continuity of care. Legend: Listed are the significant differences in patient satisfaction between participants that did experience some form of discontinuation of care, including change in diagnosis, medical therapy or discontinuation in general. In every instance, the group that did not experience any discontinuity, showed a significantly higher satisfaction.

## Data Availability

The dataset generated and analyzed during this study is not publicly available but is available from the corresponding author on reasonable request after obtaining ethics committee approval.

## Abbreviations

arcT: Autoinflammation reference center Tuebingen
ARDIS: Arthritis and Rheumatology Documentation and Information System; axaris - software & systeme GmbH
COVID-19: Coronavirus disease 2019
DEGS1: Studie zur Gesundheit Erwachsener in Deutschland
EQ-5D-3L: EuroQol 5 dimensions 3 levels
EQ-5D-5L: EuroQol 5 dimensions 5 levels
ERA: Enthesitis related arthritis
EULAR: European Alliance of Associations for Rheumatology
HR-QoL: Health-related quality of life
INDIRA: Center for Interdisciplinary Clinical Immunology, Rheumatology and Autoinflammatory Diseases
JAK: Janus kinase
JIA: Juvenile idiopathic arthritis
NSAIDs: Nonsteroidal Anti-inflammatory Drugs
PA: Polyarthritis
PGA: Physician Global Assessment
PReS: Paediatric Rheumatology European Society
OA: Oligoarthritis
QoL: Quality of life
SCTD: Systemic connective tissue disorder
SD: Standard deviation
TTP: Tuebingen Transition Program
VAS: Visual analogue scale
WHO: World Health Oragnization
