# Supplementary material for: Health-related quality of life, continuity of care and patient satisfaction: long-term outcomes of former patients of the Tuebingen Transition Program (TTP) – a retrospective cohort study
Source: Pediatr Rheumatol Online J. 2022 Dec 27;20:121. doi: 10.1186/s12969-022-00776-6 (PMC9794404; doi:10.1186/s12969-022-00776-6)
Supplement: Supplementary file 1 — Additional file 1. Supplement 1. Comparison of basic demographic and clinical characteristics between responsive and non-responsive cohort. Legend: Listed are some key demographics and clinical data of the respondents compared to the non-respondents of this study. Overall, respondents were slightly younger, and subgroups of JIA were more common as well as treatment with biologicals. Supplement 2: Significant differences between participants concerning continuity of care. Legend: Listed are the significant differences in patient satisfaction between participants that did experience some form of discontinuation of care, including change in diagnosis, medical therapy or discontinuation in general. In every instance, the group that did not experience any discontinuity, showed a significantly higher satisfaction. [file 12969_2022_776_MOESM1_ESM.docx]

**Supplement 1:** Comparison of basic demographic and clinical characteristics between responsive and non-responsive cohort

| **Variable** | **responding cohort** | **non-responding cohort** |
| --- | --- | --- |
| Age in years, median (range) | 24.1 (19.1–40.5) | 26.4 (18.5–40.2) |
| Sex, m:f, n (%) | 25:60 (29.4:70.6) | 65:145 (30.9:69.1) |
| Diagnosis at time of transfer, n (%)  Juvenile Idiopathic Arthritis (JIA), Systemic onset  JIA, Oligoarticular (extended)  JIA, Oligoarticular (persistent)  JIA, Polyarticular (RF negative)  JIA, Enthesitis-related  JIA, Psoriatic  JIA, sonstige  Autoinflammatory Disease  Connective tissue disease  Other | 2 (2.4)  3 (3.5)  10 (11,8)  23 (27,1)  14 (16,5)  5 (5,9)  3 (3,5)  6 (7,1)  8 (9,4)  11 (12,9) | 7 (3.4)  9 (4.3)  23 (11.1)  20 (9.7)  19 (9.2)  10 (4.8)  13 (6.3)  32 (15.5)  31 (15.0)  40 (19.3) |
| Medical therapy at time of transfer, n (%)  NSAID therapy  Basic therapy  Biologicals and JAK-inhibitors  Steroids  Multitherapy | 30 (35.3)  24 (28.2)  28 (32.9)  1 (1.2)  1 (1.2) | 88 (39.8)  60 (27.1)  45 (20.4)  14 (6.3)  14 (6.3) |
| PGA at time of transfer, mean (SD) | 1,01 (1,15) | 1,30 (1,67) |
| Utilization of the TTP in general  Age at first visit in the TTP in years, median (range)  Age at transfer in years, median (range)  Duration of treatment in the TTP in years, median (range)  Number of visits in the TTP, median (range) | 15.2 (11.2–18.7)  18.5 (17.0–20.7)  3.4 (0.4–7.1)  7.0 (2–18) | 15.4 (8.5–22.1)  18.5 (17.0–24.7)  3.3 (0.3–10.0)  6.0 (2–17) |

Legend:

*Listed are some key demographics and clinical data of the respondents compared to the non-respondents of this study. Overall, respondents were slightly younger, and subgroups of JIA were more common as well as treatment with biologicals.*

***Supplement 2****: Significant differences between participants concerning continuity of care*

| **Dependent variable**  Satisfaction with... | **Independent variable** | **eta-coefficient** | **p-value** |
| --- | --- | --- | --- |
| the TTP in general | Change in diagnosis: no | 0.266 | 0.006 |
| knowledge about one’s disease | Discontinuation of care after transfer: no | 0.419 | 0.012 |
| knowledge about one’s therapy and possible side-effects | Change in medical therapy: no | 0.503 | 0.004 |
| knowledge about one’s therapy and possible side-effects | Discontinuation of care after transfer: no | 0.441 | 0.008 |

Legend:

*Listed are the significant differences in patient satisfaction between participants that did experience some form of discontinuation of care, including change in diagnosis, medical therapy or discontinuation in general. In every instance, the group that did not experience any discontinuity, showed a significantly higher satisfaction.*
